# Fractal Structure in Silica and Composites Aerogels

**DOI:** 10.3390/gels7010001

**Published:** 2020-12-26

**Authors:** Thierry Woignier, Juan Primera, Adil Alaoui, Philippe Dieudonne, Laurent Duffours, Isabelle Beurroies, Sylvie Calas-Etienne, Florence Despestis, Annelise Faivre, Pascal Etienne

**Affiliations:** 1 Institut Méditerranéen de Biodiversité et d’Ecologie Marine et Continentale (IMBE), Aix Marseille Université, CNRS, IRD, Avignon Université, UMR CNRS 7263, 13397 Marseille, France; 2 IRD UMR 237-Campus Agro Environnemental Caraïbes-B.P. 214 Petit Morne, 97232 Le Lamentin, Martinique, France; 3 Departamento de Fisica, FEC, LUZ, 4011 Maracaibo, Venezuela; juan.primera2009@gmail.com; 4 Departamento de Ciencias Agrícolas, Facultad de Ingeniería Agrícola, Universidad Técnica de Manabí, Lodana 130105, Ecuador; 5 Faculté des Sciences et Techniques de Tanger, B.P. 416, 90000 Tanger, Morocco; pr_alaoui@yahoo.fr; 6 Laboratoire Charles Coulomb, Université Montpellier 2, Place E. Bataillon, CEDEX 5, 34095 Montpellier, France; philippe.dieudonne-george@umontpellier.fr (P.D.); sylvie.etienne@umontpellier.fr (S.C.-E.); florence.despetis@umontpellier.fr (F.D.); annelise.faivre@umontpellier.fr (A.F.); pascal.etienne@umontpellier.fr (P.E.); 7 PrimeVerre, 34090 Montpellier, France; laurent.duffours@primeverre.com; 8 MADIREL, Aix-Marseille Université (Saint Jérôme), CNRS, AVE Escadrille Normandie Niemen, 13013 Marseille, France; isabelle.beurroies@univ-amu.fr

**Keywords:** aerogel, composite aerogel, fractal, SAXS, microstructure

## Abstract

Silica aerogels are known to be materials with exceptional characteristics, such as ultra-low density, high surface area, high porosity, high adsorption, and low-thermal conductivity. In addition, these unique properties are mainly related to their specific processing. Depending on the aerogel synthesis procedure, the aerogels texture can be tailored with meso and/or macroporosity. Fractal geometry has been observed and used to describe silica aerogels at nanoscales in certain conditions. In this review paper, we describe the fractal structure of silica aerogels that can develop depending on the synthesis conditions. X-ray and neutron scattering measurements allow to show that silica aerogels can exhibit a fractal structure over one or even more than two orders of magnitude in length. The fractal dimension does not depend directly on the material density but can vary with the synthesis conditions. It ranges typically between 1.6 and 2.4. The effect of the introduction of silica particles or of further thermal treatment or compression of the silica aerogels on their microstructure and their fractal characteristics is also resumed.

## 1. Introduction

Aerogels have drawn increasing interest in different fields ranging from fundamental physics research to applications as specific materials. Silica aerogels are unique materials due to their very peculiar physical properties, such as very low sound velocity [1], large specific surface area [2], and low thermal conductivity [3]. It has also been proposed that they can develop a fractal structure [4,5]. These properties and features are essentially due to the very large pore volume of these materials, which can be tailored up to 99% by the sol-gel synthesis conditions [6] (i.e., the siloxane monomer content, pH), but which can also be modified by sintering [7] or compression processing [8].

There are currently many applications of aerogels, such as catalysts [2], insulators [3], sensors [9] environmental [10] and biomedical applications [11,12], etc., and the potential uses of these materials are even larger if one considers the aerogel as a precursor. Through heat treatments, the silica aerogels can indeed be sintered into silicate glasses and glass ceramics [13,14]. However, these porous materials can also be used as a matrix for the synthesis of multi-phase materials, doped materials, or composites. The large pore volume can be used as a host to incorporate other chemical species and form a two-phase material. The chemical species are initially processed in liquid form but can be dried after pores filling [14,15].

Aerogels are also interesting in theoretical research. They are ideal materials to analyze the change in their physical properties as a function of their structure which can be studied experimentally over an exceptionally large range of porosity from 0% to 99%. Moreover, molecules confinement in the nanopores of these materials can lead to interesting specific behaviors.

Aerogels main physical properties such as mechanical properties, permeability, transparency, insulation, etc. are mainly govern by their microstructure [16,17,18,19,20,21,22,23,24,25,26,27,28,29,30], which has been extensively studied during the past decades by different scattering techniques (SAXS, SANS, light scattering [31,32,33,34,35,36,37,38,39,40,41,42]). The silica aerogel microstructure is generally described as a fractal network at length scales ranging from 1 to 100 nm. The fractal structure is explained as the result of a special aggregation mechanism. The silica beads (≈ 1nm) build clusters with a compactness characterized by a fractal dimension Df. The clusters spatial extent and their fractal dimension are strongly dependent on the synthesis conditions, and especially on the pH of the gelling solution [31,32,41].

For a porous material, the fractal range spans between two limits [32,43,44]. The lowest fractal dimension limit is the size of the primary particles from which the fractal is built up. The upper limit is the size of the largest clusters. The fractal dimension Df quantifies the change of the mass of solid as a function of the observation scale. Df gives information on the cluster compactness and sometimes on the aggregation mechanism. Several models like diffusion limited cluster aggregation (DLCA) or reaction limited cluster aggregation (RLCA) have been proposed in the literature to account for the observed Df changes in relation with the aggregation mechanisms [45,46].

Composite silica aerogels [47,48] have been synthesized by adding silica soot (aerosil) in the gelling solution, with the consequence of tailoring the pore sizes. One relevant question which has been raised in the literature is consequently: “how does the presence of these silica particles disturb the organosilane’s aggregation process and affect the fractal microstructure?”. Composite aerogels with increasing silica soot content were prepared in order to understand the transition from a fractal to a non-fractal aerogel. For that, the network structure has been characterized in the length scale ranging from 1 to 1000 nm (micro, meso and macroporosity).

Heat treatments [7,49] provide another route to modify the fractal geometry. At high temperature, as sintering proceeds, the particle sizes, the compactness, and the cluster sizes vary. It has also been quoted in the literature that aerogels can be compacted by an isostatic pressure [8,50,51]. The induced shrinkage volume has been shown to be related to microstructure evolution (fractal feature changes) induced by pressure densification.

As microstructure is the key parameter of many aerogel properties, in this short review, we try to resume different ways to control silica aerogels microstructure and discuss their respective influence. We will show the effect of synthesis parameters, such as the organosilane concentration, pH of the solution, and the effect of silica particles addition, but also the effect of porous collapse induced either by aerogels sintering or by aerogels compression.

## 2. Literature Results Synthesis

### 2.1. Fractal Geometry as Obtained by SANS, SAXS and USAXS Measurements

**Figure 1:**
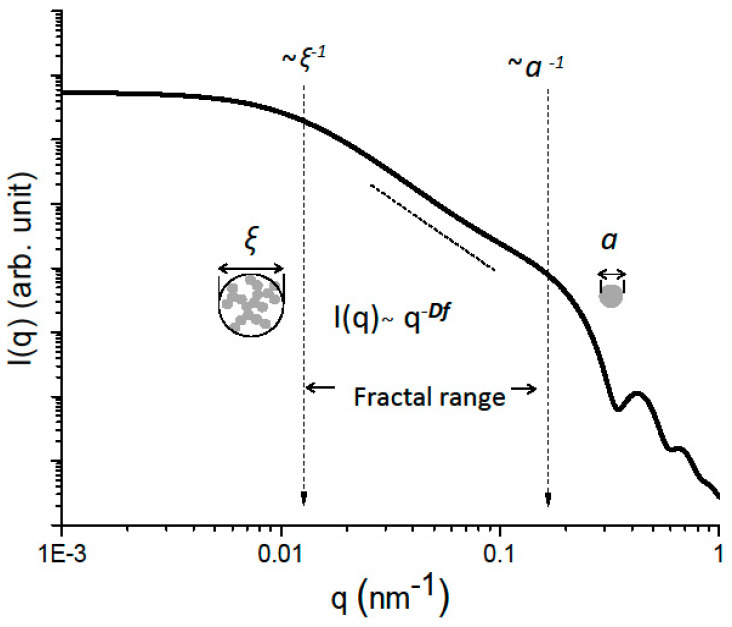
Typical I(q) as measured by SAXS, SANS or USAXS for a fractal aerogel.

A shown in the literature [32,33,43,44], small angle X-ray or neutron scattering experiments can provide information on three different aspects of aerogels fractal geometry: the mean size of the fractal clusters (ξ), the mean size of the primary particles (a) which stick together to build the cluster, and the fractal dimension Df which expresses the clusters compactness. These structural features are related to different length scales of the scattering patterns as shown in Figure 1. The power law allowing to describe the linear behavior in the intermediate q range of I(q) plotted in a log-log scale is associated to −Df. The position of the two cross-overs is respectively related to the inverse of the cluster size ξ and the inverse of the particle size “a”.

The power law behavior is however sometimes limited (especially for small values of −ξ/a ratios as will be explained latter), and Df is not easily accessible. In this case, it has been shown that SAXS data can be fitted with the relation (1) [48,49], as proposed by Texeira et al. and Chen et al. [43,44] when considering an assembly of spherical particles aggregated to form clusters having disordered fractal geometry:

I(q)(1) = Aρ^2^ξ^2^[Γ(Df + 1) sin (Df − 1) arctan (qξ)]/[(1 + q^2^ξ^2^) ^(Df−1)/2)^ (Df − 1)qξ] (1)

where A is a constant depending on the square of the average scattering length and Γ represents the gamma function. This equation (1) has been obtained by considering that the finite size of the fractal aggregates, which may induce correlations, may be represented by a scaling function assumed to be an exponential (exp(−r/ξ)), where r is the measured dimension.

Fitting the experimental curve with Equation (1) provides an estimate of the fractal dimension Df and of the average correlation length ξ Fitting data from Guinier to fractal regimes. The primary particle size a is deduced from the crossover between fractal and Porod’s regimes.

### 2.2. Aerogels: Fractal Structure Materials?

One may be surprised by such a provocative title. However, in the literature [32,33,34,35,36,37,38,39,40,41], different conclusions have been drawn about the fractal nature of silica aerogels. Some authors argue that these materials are not fractal or that a fractal structure would be limited to a small-scale area. These inconsistences could be explained by considering the different synthesis conditions, such as pH, temperature, and gel aging duration before supercritical drying. Another explanation may be that the observed fractal domain is too small, typically less than an order of magnitude in length. Under these conditions, a correct determination of Df is difficult and one can question the meaning of a fractal structure over such a small length scale [44].

In order to clarify this issue, it is necessary to check experimentally various silica aerogels in order to define the conditions under which a fractal structure can be observe in these materials [32].

The mass M of a fractal object of size L is given as:

M α L^Df^ (2)

Thus, its density is:

ρ(L) α L^Df−3^ (3)

**Figure 2:**
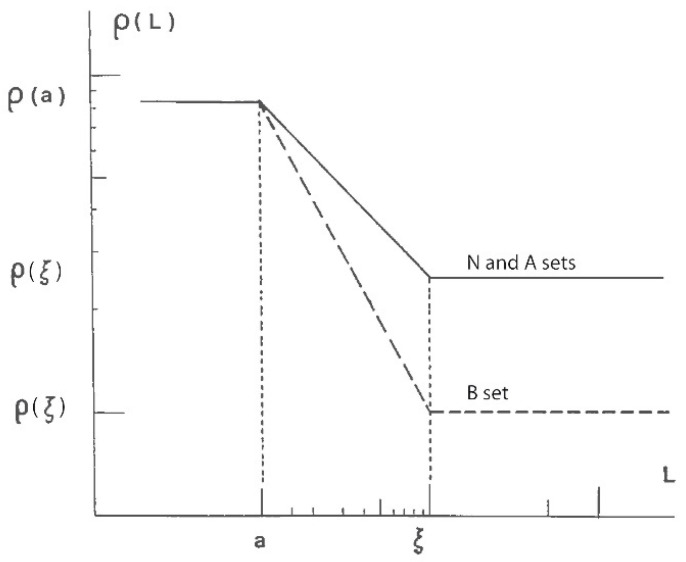
Evolution of the bulk density in a fractal material (issued from [32]).

Real materials have usually a fractal structure over a limited range of lengths (see Figure 2). The lowest limit is given by the size (a) of the primary particles constituting the material. At lengths smaller than (a) the structure is not fractal and the density ρ(a) does not depend on the analysed length scale. It is a constant, equal to the skeletal density.

At the other extreme, the fractal structure of the material is limited to a length ξ above which the material structure can be considered as homogeneous. Therefore, at this scale, the density approaches the bulk density ρ (Table 1).

By applying the scaling law (3) to ξ and a, one obtains:

ρ(ξ) = ρ(a) × [ξ/a]^Df−3^ (4)

reflecting the change in the apparent density of the fractal aggregate of length ξ with ξ/a and Df (Figure 2).

In order to make it so that the analysis of the scattering results has a physical meaning in terms of fractal structure, it is necessary for the ξ/a ratio to be large enough. We postulated that a good criterion to determine the value of Df with sufficient accuracy is that at least a decade should separate ξ and (a) [32]. If the ratio ξ/a is at least equal to 10, then applying Equation (4) indicates that ρ(ξ) will be low.

With a fractal ratio equal to 10, a skeletal density in the range 1.8–2 g·cm^−3^ as measured by He pycnometry [52] and a fractal dimension close to 2.2–2.4 for neutral and acidic aerogels and close to 1.8 for basis aerogels [33], we can estimate the value of the aerogels bulk density using Equation (3).

For base catalyzed aerogels, the bulk density would be ρ < 0.13 g·cm^−3^. As a consequence, for base catalyzed aerogels, highly tenuous materials need to be synthetize to present a clear fractal structure (Figure 2). The materials are close to the limit of the mechanical stability.

For neutral and acidic aerogels, the bulk density would be ρ < 0.35 g·cm^−3^.

The rough values can be considered as an estimate of the density range in which fractal geometry can be expected. Table 1 will consequently be useful to select the fractal “candidates”.

### 2.3. Influence of the Alkoxide Content and pH on Fractal Features

The influence of the conditions of silica gels synthesis on the final aerogel’s fractal structure has been analyzed in the literature. For example, alcogels were prepared by hydrolysis and polycondensation reactions of tetramethoxysilane (TMOS) [37]. The TMOS was dissolved in various amounts (2–46 volume %) to adjust the oxide content of the sol (and consequently the final bulk density of the material). The solutions were hydrolyzed under neutral, basic (ammonia, 5 × 10^−2^ N), or acidic (nitric acid, 10^−4^ N) conditions. N is the gram equivalent weight of a solute per liter of solution. The molar ratio H_2_O/TMOS is 4. Aerogels were obtained by supercritical drying treatment in an autoclave (305 °C, 13 MPa) [38,39]. The samples were labeled Ax, Nx, and Bx for acid, neutral, or base catalysis conditions, respectively, and x referred to the TMOS volume concentration in the solution. The density range varies between 0.02 and 0.5 g·cm^−3^ (see Table 1). The bulk density of the samples was calculated by weighing aerogel cylinders (5 samples mean value) of know dimensions and the standard deviation is 10^−2^ g·cm^−3^.

**Figure 3:**
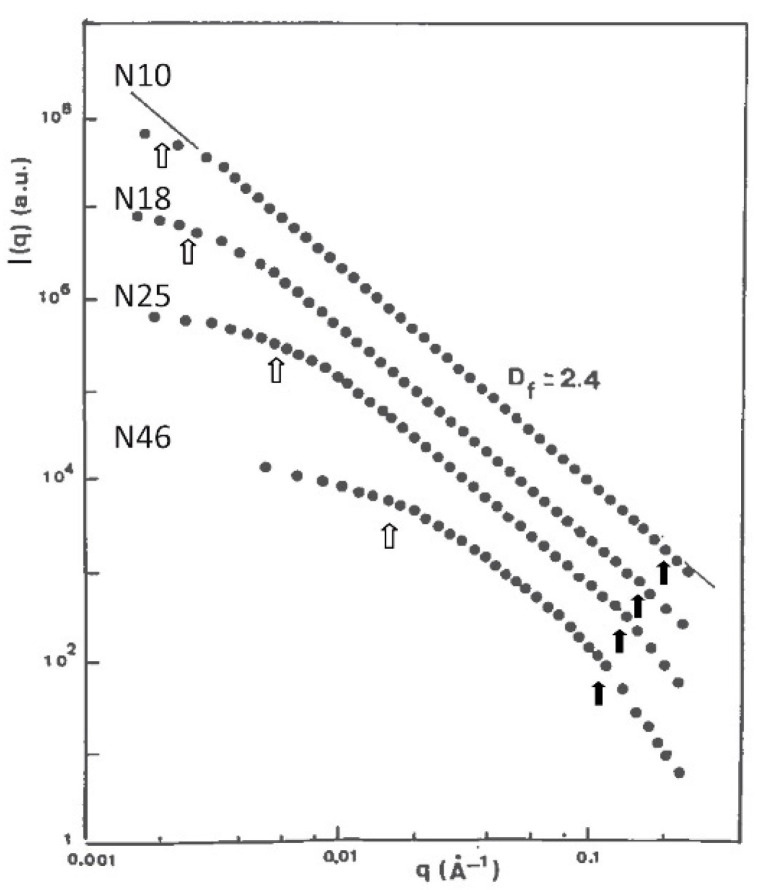
I(q) as measured by SANS for the neutral set (from [32]).

**Figure 4:**
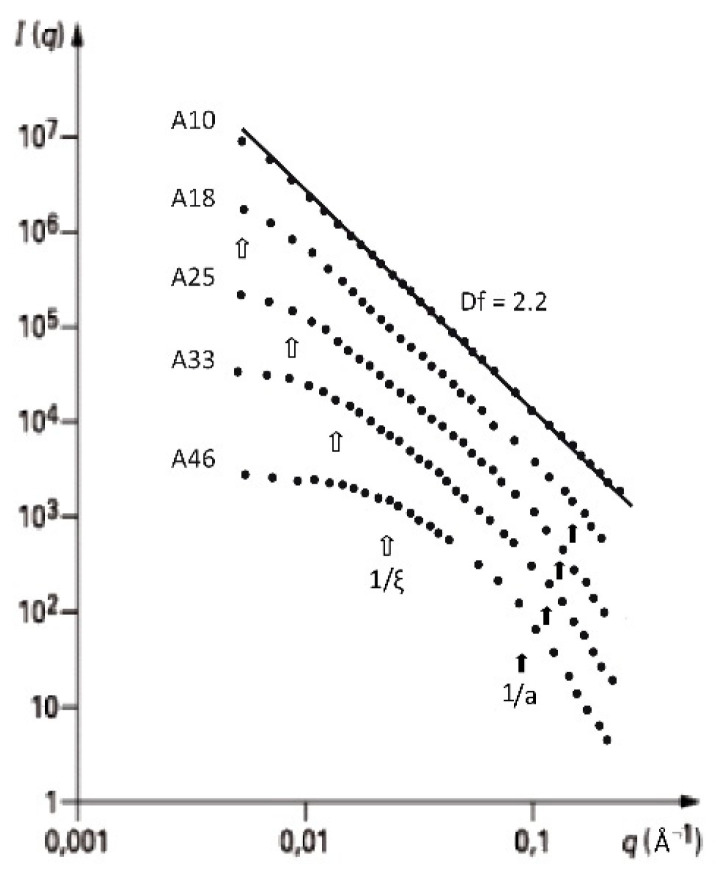
I(q) as measured by SANS for the acid set (from [32]).

**Figure 5:**
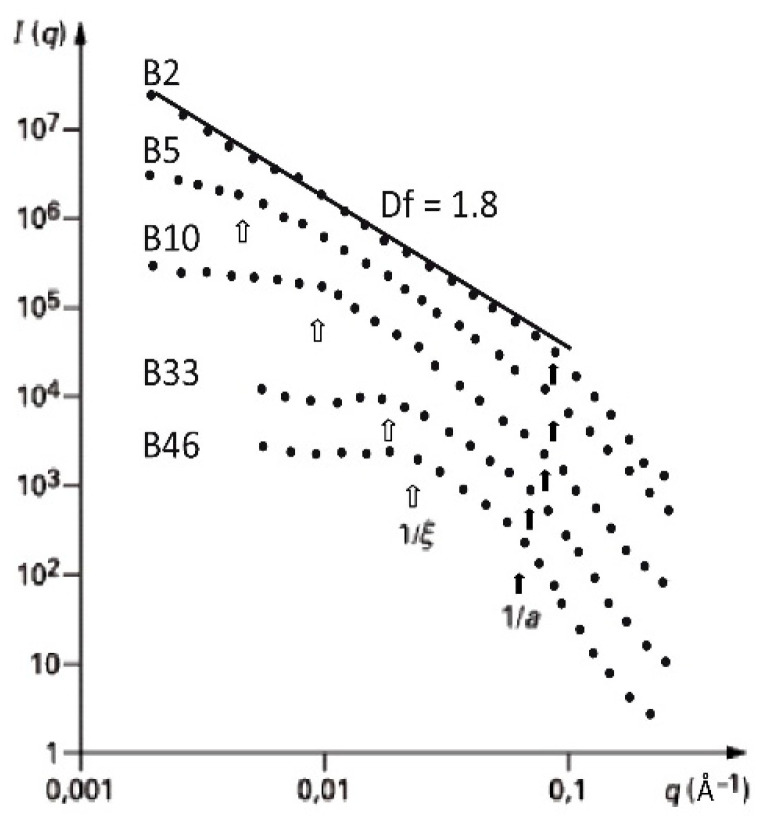
I(q) as measured by SANS for the base set (from [32]).

Figure 3, Figure 4 and Figure 5 show the evolution of the SANS intensity I(q) versus q measured in aerogel samples prepared with different TMOS concentration and for three kinds of hydrolysis conditions (neutral, acid, and basic catalysis) respectively.

For the three catalysis conditions, increasing the organosilane content of the sol shortens the fractal domain (linear dependence of I(q) in log-log plot). Moreover, we will further show that the microstructures of neutral and acidic catalysed aerogels are very different to that obtained with a base catalysis.

Going deeper in the analysis of Figure 3, it must first be underlined that I(q) α q^−Df^ is observed for the N10 sample over almost two orders of magnitude in q. Then, for the lightest samples, fractal geometry extends down to the smallest length scale probed in this experiment with value of Df = 2.4. For sample N46, the departure of I(q) from the q^−2.4^ dependence at large q indicates the presence of particles with gyration radii of 10 Ǻ. For this sample, above ~0.15 Ǻ^−1^_,_ I(q) found to be nearly proportional to q^−3^. This power law suggests fuzzy particles with a fractal surface [36,37]. The structure at that scale can be modified by oxidation at 500 °C. Remaining -CH_3_ groups are removed at 500 °C and new siloxane bonds are created [6,14]. After such a treatment, one observes q^−4^ dependence of I(q) at large q, demonstrating that oxidation smoothens the surface of the particles.

Same kind of conclusions can be drawn from Figure 4. For this acid catalyzed silica aerogels, the value of Df is in the range of 2.2–2.3. As expected, the range of scale at which the material is fractal depends on the apparent density of the aerogel. For sample A10, the fractal domain covers almost two orders of magnitude. When the TMOS content of the sol increases, ξ/a decreases, and for the densest material (A46), it becomes difficult to highlight clearly a fractal structure. It can only assume that this material is fractal by analogy and continuity with the other samples, and only one a small length.

Turning now to base catalyzed silica aerogels presented in Figure 5 we can observe that the size of the primary particles is bigger around 10–20 Ǻ weakly dependent on the aerogel density [33]. Furthermore, the fractal domain is rather limited and only extend over one order of magnitude for the lightest samples B2, B5 and B10. For the former, the power-law behavior extends down to the smallest accessible q-values. As explained above (see Section 2.4. and Figure 2) as Df is low (1.8), it will be necessary to synthesize extremely tenuous materials in order to have a ξ/a ratio higher than 10.

In summary, we can say that a fractal structure can be clearly observed in certain silica aerogels depending on the conditions of the sol-gel process:

-For acid and neutral catalysed samples, the radius (a) of the primary particles is smaller than 1nm while base catalyzed aerogels show a higher primary particles size (˃1.5 nm).

-The fractal dimension is found to be close to 2.2–2.4 for acid and neutral conditions, 1.8 for basic catalysis, and almost doesn’t depend on the aerogel density for the same catalysis conditions.

**Figure 6:**
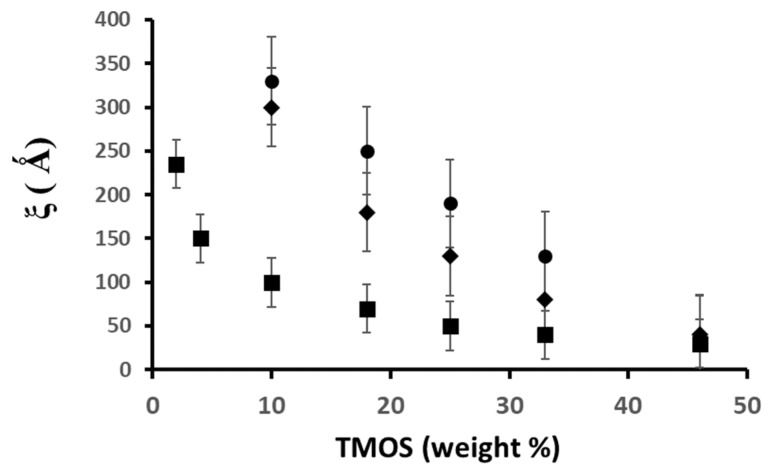
Evolution of ξ versus TMOS concentration for the different catalysis conditions:.base (■), neutral (♦), acid (●).

-The influence of the sol TMOS concentration on ξ is summarized in Figure 6 for the different catalysis conditions. These results demonstrate that aerogels with a fractal geometry over a length scale larger than one decade can be prepared. However, only very light aerogels (prepared with a low TMOS concentration, i.e., B2, B5, and B10) exhibit a very clear fractal structure. The elementary particles can be rough or smooth, and their size strongly depends on catalysis conditions.

These results clearly indicate that the specific modifications in the sol-gel process obviously induce very different aggregation conditions, leading to different structures. In particular, in the case of base catalysis, it appears that diffusion-limited cluster–cluster aggregation model can probably explain the gel formation.

As already said, for each type of catalysis, the fractal dimension is independent of the aerogel density, and more precisely of the TMOS concentration of the sol. This result suggests that, before gelling, the aggregates grow independently of each other by a mechanism related to the condition of catalysis. The gelling occurs when these clusters stick together to form a continuous network. The size of the clusters is limited by the proximity of the neighboring clusters and therefore by their density in number.

It is often assumed in the literature that the value of Df only depends on the aggregation process. However, discrepancies between the experimental and calculated (using classical aggregation models) values of Df indicate that this might not be so simple. A gel forms when fractal clusters aggregate to establish a solid network. However, unaggregated clusters continue to stick to the percolating network well after the gelation point (aging). Aged gel finally consists of entangled clusters wetted by the liquid solvent. The supercritical drying process induces a restructuring phenomenon (syneresis) [5], as observed from the occurrence of shrinkage. The fractal dimension is measured on the final structure of the aerogel, which also depends on the syneresis during the supercritical drying [33].

### 2.4. Influence of the Addition of Silica Particles on the Fractal Features 

In the literature [47,48], it is shown that the addition of pyrogenic silica such as "aerosil" in the organosilane solution before gelation, favors the formation of macropores. In these studies, fumed silica (aerosil OX50, Degussa) was used. It is a hydrophilic silica powder with a specific surface area of around 50 m^2^.g^−1^. The aerosil powder was added to the hydrolyzed solution of tetraethoxysilane (TEOS). The aerosil weight percentage (reported relative to the total silica weight coming from TEOS + aerosil) ranged between 0 and 70%. The pH of the sol was then adjusted to 4.5, which leads to gelation within a few minutes. The aerogels were labelled as CA y, where y is the aerosil content in weight percent. These different samples covered bulk densities within the range 0.25–0.4 g·cm^−3^ [48].

**Figure 7:**
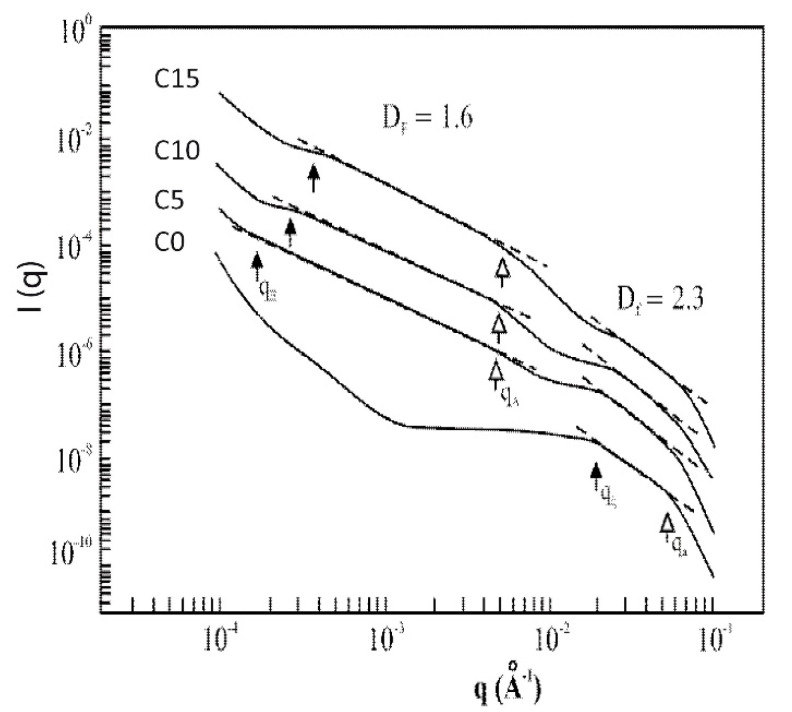
I(q) as measured by USAXS for the composite set (C0–C15) (from [48]).

It was shown that the aerosil addition affects the aggregation mechanism, the aerogel structure, and the pore-size distribution. The data given in Figure 7 show the change in the scattering intensity I (q) (as measured by USAXS) for the aerogel composite set as a function of the aerosil concentration.

The position of the two crossovers (at q_ξ_ and q_a_) for the sample C0 are related respectively to the inverse of the cluster size (ξ) and the inverse of the particle size (a). The slope between the two crossovers corresponds to −Df, as previously explained. We can deduce that (a) is close to 10 Å, ξ close to 50 Å and Df close to 2.3. These results are in good agreement with those previously measured on classical aerogels issued from organometallic compounds (see Section 2.2.) [32]. The intensity increase as the q value decreases (below 10^−3^ Å^−1^) is the signature of macroporosity, the typical length scale of which is around a few hundred nanometers.

With only 5% of aerosil addition, the scattered intensity is strongly affected. The curves measured for samples C5, C10 and C15 exhibit the same trends. Besides the high q (>10^−2^ Å^−1^) section of the curve previously described, there is a broad linear behavior between almost 10^−4^ to 5 × 10^−3^ Å^−1^. The cross over at q = 5 × 10^−3^ Å^−1^ can be associated to the aerosil particle size (≈200 Å). The linear behavior at low q is interpreted as a fractal structure issued from the soot particles aggregation. The fractal dimension of this structure (Df = 1.6) is in agreement with a DLCA aggregation process [45,46].

**Figure 8:**
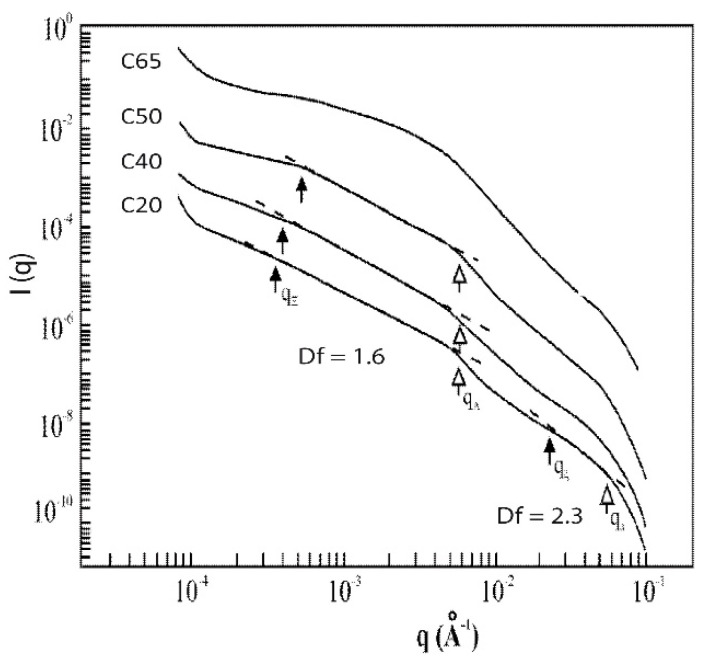
I(q) as measured by USAXS for the composite set (C20–C65) (from [48]).

For composites with a higher aerosil content (Figure 8), the crossover corresponding to the aerosil particles size is more pronounced as the aerosil content increases. Among differences, one can first note that the polymeric network geometry is progressively destroyed by the presence of the aerosil particles. It is not possible to observe the typical curve of the polymeric gel in the 10^−2^–10^−1^ Å^−1^ q range. Secondly, the extent of the fractal range of the aerosil cluster also decreases with the increase of the aerosil content and the sample C65 is no longer fractal.

### 2.5. Influence of the Aerogel Sintering on Fractal Features

As explained in the introduction, aerogels can be sintered and transformed into dense silica glasses by a controlled heat treatment [49]. In the case of neutrally reacted aerogels N18, after a preliminary oxidation heat treatment (12 h at 300 °C in air), the porosity is progressively removed with time at temperatures higher than 1000 °C and sintered samples are obtained with bulk densities in the range of 0.16 g·cm^−3^ to 0.72 g·cm^−3^. We can expect significant changes in the microstructure over this large density range. In a previous study [53], cylinders of neutrally reacted aerogels N18 were sintered for increasing durations up to a chosen density.

**Figure 9:**
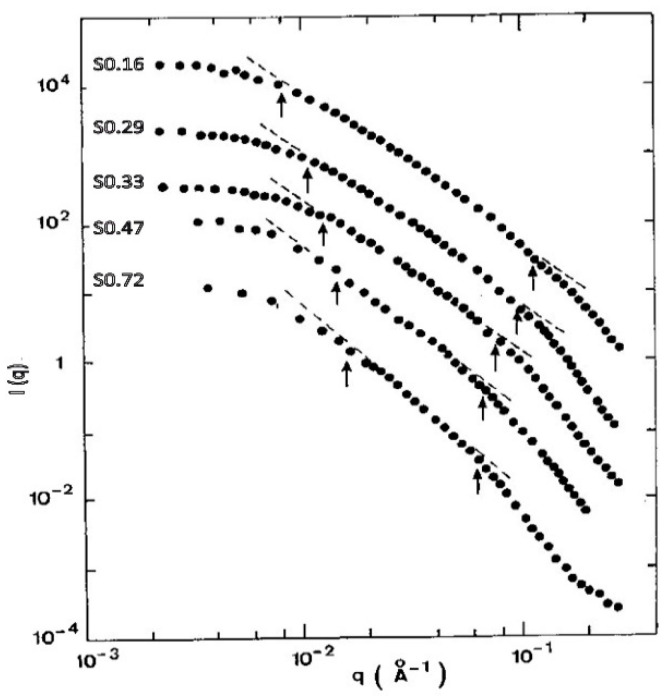
I(q) as measured by USAXS for the N18 sintered aerogels (from [53]).

**Figure 10:**
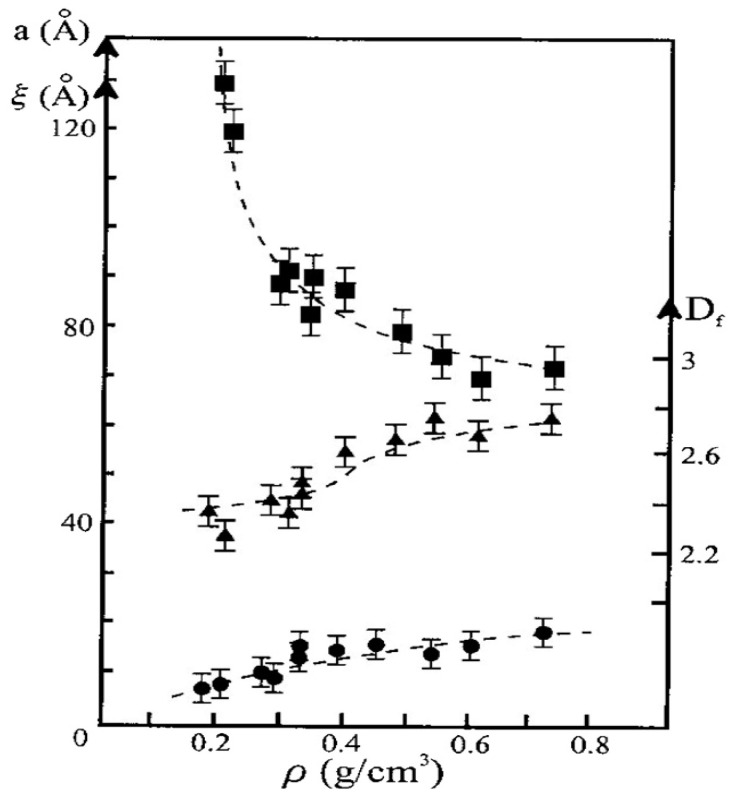
ξ (squares), a (circles) and Df (triangles) evolution versus the bulk density for the sintered aerogels set (from [53]).

The SAXS intensities for five of these samples are shown in Figure 9, labelled “SXXX”, XXX being the density after sintering. The values of ξ, a, and Df determined from the SAXS data of all the sintered samples are summarized in Figure 10.

### 2.6. Influence of the Compaction Process on Fractal Features

Densification by isostatic compression can be processed using mercury porosimetry on outgassed aerogels. As mercury cannot penetrate the pores, the aerogel has been showed to be isostatically compressed [8,50,51]. Owing to its compliance, the sample deforms and the residual volume strain, when the pressure is released, corresponds to the volume collapse. The aerogel may be compressed up to a chosen pressure varying from 0.1 to 200 MPa. After depressurization, the irreversible volume shrinkage measured from the mercury level using a cathetometer allows the bulk density to be calculated.

**Figure 11:**
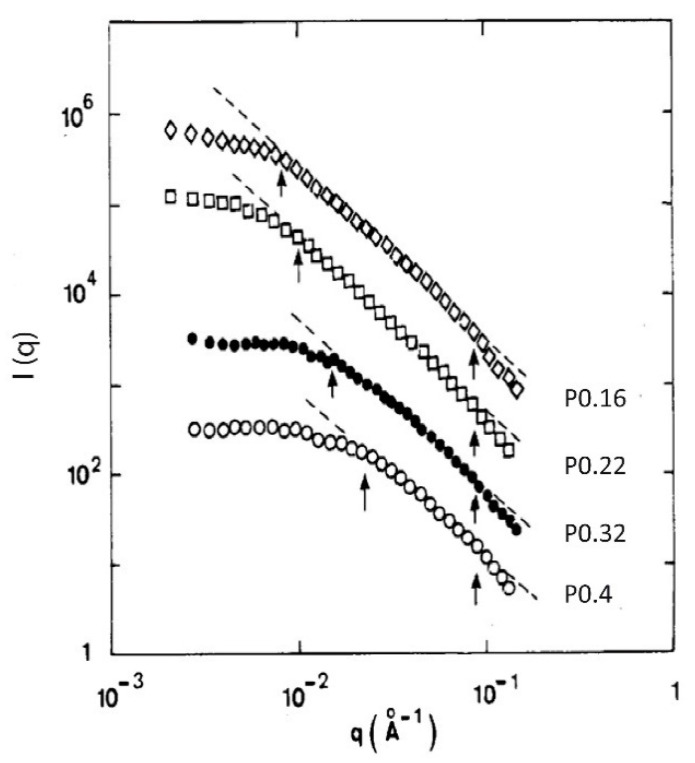
I(q) as measured by SAXS for the N18 compressed aerogels set (from [53]).

**Figure 12:**
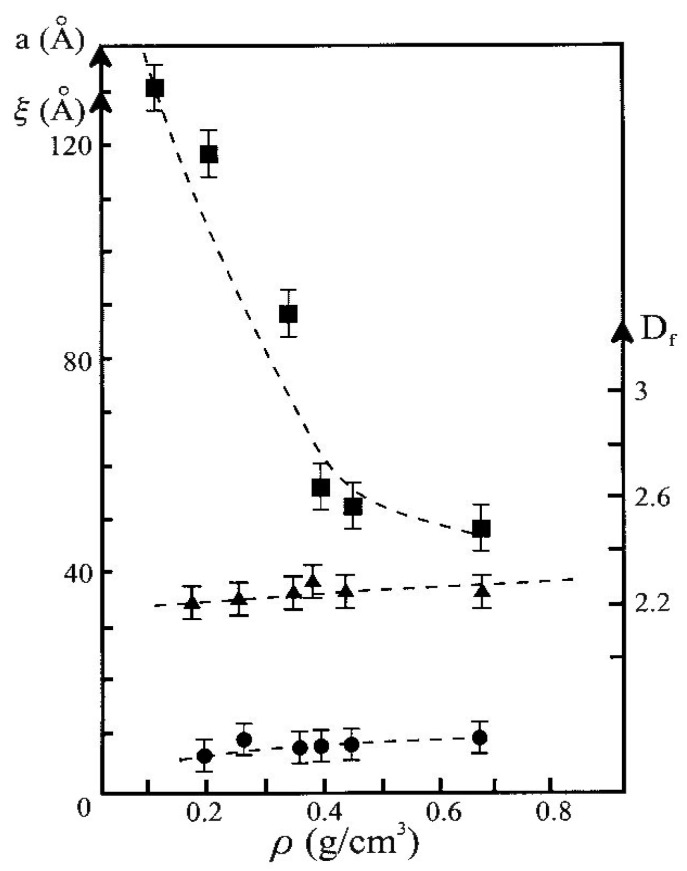
ξ (squares), a (circles) and D_f_ (triangles) evolution versus the bulk density for the N18 compressed aerogels set (from [53]).

The aerogel compaction by isostatic pressure was investigated on compacted samples with densities in the range of 0.16–0.7 g·cm^−3^. Neutrally reacted aerogel N18 of density ρ = 0.16 g c^3^ were compacted by increased pressure up to a defined density. The SAXS intensities measured in five samples, labelled “PXXX”, XXX being the density after compression, are shown in Figure 11. The values of ξ, a, and Df, determined from the SAXS data of all the compressed samples are summarized in Figure 12.

One notices that the size of the primary particles and the fractal dimension Df are practically unchanged with aerogel compaction, as long as a fractal description remains meaningful. On the contrary, the correlation length ξ first strongly decreases when the density increases up to 0.35 g·cm^−3^, then it remains constant for higher pressures while density continues to increase. The accuracy of the measurement mainly depends on the extent of the fractal range. The value of (a) corresponding to aerogels which exhibit a fractal structure on a short length scale and for which a straight line cannot easily be drawn, is difficult to appreciate making it a rough estimate rather than a precise value. As the densification proceeds, the (a) value remains constant and close to 10 Å. There is very small increase in Df with compaction, but it is almost negligible and in does not reach the value of 3 as measured for aerogels densified by sintering. Sintering changes the texture at the microscopic scale smoothing the surface, eliminating the micro pores. Compression transforms the material at the macroscopic scale but does not modify the microstructure.

## 3. Discussion

The fractal dimensions deduced from the results presented above are different for acid, neutral (2.2–2.4), and especially for base catalyzed aerogels (1.8). We can consequently suppose that the aggregation mechanism during the sol-gel process is different: a RLCA mechanism for acid and neutral sets and a DLCA mechanism for the base set. However, no chemical justification has been given in the literature to confirm these different aggregation mechanisms. Moreover, the higher fractal dimension could also be due to change in the microstructure happening during the aging or drying process which could increase the connectivity. Table 1 shows that, for the same TMOS content in the sol, the bulk density of aerogels A and N are twice the bulk density of B, indicating a larger shrinkage during the supercritical drying [54]. Therefore, the fractal dimension measured in the aerogels is not the signature of the aggregation process one its own. Df depends on the aggregation mechanism during sol/gel process, but also on the syneresis during aging and on the shrinkage during supercritical drying. This shrinkage is indeed large for acid and neutral aerogels and limited for the basic sets [54].

A new particularly interesting result concerns the USAXS data measured in these polymeric classical aerogels (Figure 7) at low q values and especially in the domain of long ranges of scale length (above 10^3^ Å), as little information is available in the literature. The increasing intensity for decreasing q values is associated to macropores with a typical length of around 10^3^ Å. Such results are not observed by the scattering techniques (SAXS, SANS, and light scattering) because of the limitations in the accessible values of q. A macroporous volume has indeed been deduced from the thermoporometry results on analogous polymeric aerogels.

When aerosil is added to create composite aerogels, for small relative concentrations in aerosil to total amount of silica (C5–C15), two different fractal domains are revealed by USAXS corresponding to length scales in the range of 10–100 Å (which is the usual fractal range for polymeric aerogel) and length scales in the range of 150–2000 Å. The data can be explained by considering that the composite aerogel structure is the sum of the classical network issued from the gelation of organosiloxane (polymeric gel) and a network issued from the aerosil particles. The aerosil particles, covered with hydroxyl groups are consequently suitable sites for the hydrolysis of TEOS molecules. For a low aerosil content, the gel formation is almost not affected by the presence of the aerosil particles giving rise to the classical fractal structure previously described (Df 2.2–2.4).

As the polymeric clusters surrounding the aerosil particles link together, a network of aerosil particles is created. At the gel point, the spatial arrangement of the large particles is characterized by its fractal dimension, Df = 1.6. Such a value of Df leads to the conclusion that the DLCA model is likely appropriated to describe the aerosil network formation. For these composite aerogels, no significant restructuring phenomenon (like syneresis) takes place after gelling, as density almost doesn’t evolve during aging and super-critical drying. Moreover, the small density change is probably due to fractal classical polymeric network rearrangement. The addition of silica particles affects the structure but also the mechanical properties of aerogels [55]. Above the percolation threshold for concentrations higher than 40%, the mechanical properties have been shown to rapidly increase with aerosil content.

Concerning the partial densification by sintering, several comments can be made. The heat treatment has two main effects: it removes the smallest pores and collapses the whole network structure. During the sintering process, densification is mainly due to viscous flow, which induces the reduction of the whole sample volume by first closing the smallest pores [7,15,49]. The sintering process increases the particles size and the fractal dimension, which is the signature of the improved connectivity in the fractal aggregates. This densification of the structure at different scales is well supported by the large strengthening and stiffening of the sintered aerogels [49].

The evolution of fractal features with isostatic compression of aerogels have shown that there are two main regimes as a function of bulk density. In the range of weak pressures, the external pressure acts on fractal clusters by reducing their size, probably due to an entanglement of fractal clusters. Such an entanglement leads to a lowering of the fractal range while the lower bound of the fractal range, particle size, remains constant. The entanglement of aggregates is favored by breaking of bonds which is in agreement with the measured reduction of the material stiffness [53]. The second regime is related to the vanishing of pores located between clusters which results in a better packing of clusters. The crossover between these regimes then depends on the details of aerogel preparation.

Table 2 summarizes the main results concerning the fractal structure of silica aerogels.

We focused here on the microstructure and porosity of aerogels obtained by rather classical routes, but different alternative approaches, such as sonocatalysis, control of the depressurization rate [56,57,58,59], etc., could also be interesting to check.

The recent literature reports on studies describing new applications area for aerogel. They are indeed good candidate for the mitigation or removal of hazardous pollutants, such as volatile organic compounds, oils and solvents, or heavy metals [60,61,62,63,64,65] from the air and water, immobilization of radioactive wastes [15,16], or greenhouses gases [66,67,68,69,70]. All these new possibilities are the consequence of the very peculiar aerogels microstructure and the ways to control it. Finally, an emerging topic in aerogel science is also the environmental assessment of processes [71,72,73,74,75,76].

## 4. Conclusions

This review resumes the small angle scattering data obtained in different sets of aerogels synthesized in acidic, neutral, or basic conditions and more specifically their fractal features. For neutral and acid aerogels, the fractal dimension is always in the range 2.2–2.4 and the radius of the particles (a) is smaller than 1 nm. As expected, the fractal length scale depends on the organosilane concentration of the sol. The fractal structure spans over almost two orders of magnitude in intensity for the lightest aerogels. The base catalysis leads to the formation of larger primary particles, with a size around 15–20 Ǻ and the fractal dimension is close to 1.8. Data obtained at very small q for composite aerogels indicate that a fractal network of aerosil particles is embedded in a fractal network of a polymeric gel. Due to enhanced mechanical properties and a more homogeneous porous structure, the composite aerogels can be used as a porous and sinterable host matrix for nuclear wastes.

After aerogels sintering at 1000 °C, SAXS results show that the fractal cluster length scale (ξ) decreases while the particle size (a) grows. The SAXS data suggests that densification is due to interfacial transformations within the cluster, which pull on the network. The densification proceeds through the coalescence of several small particles into a larger one. Df appears rather constant in the initial stage of the sintering and then to increase toward 3. This increase indicates clusters densification related to particles coalescence. On the contrary, after densification by compression, the restructuring is due to a new spatial arrangement of the clusters, which interpenetrate under pressure. Their periphery is modified but their internal structure remains unaffected. Df and (a) are indeed almost constant after compression.

## Figures and Tables

**Table 1:** Aerogel bulk density versus the TMOS content for the base, neutral and acidic catalysis.

TMOS (vol. %)	Bulk Density (g·cm^−3^) B Set	Bulk Density (g·cm^−3^) N Set	Bulk Density (g·cm^−3^) A Set
46	0.22 ± 0.01	0.31 ± 0.01	0.42 ± 0.01
33	0.17 ± 0.01	0.22 ± 0.01	0.36 ± 0.01
25	0.12 ± 0.01	0.16 ± 0.01	0.3 ± 0.01
18	0.09 ± 0.01	0.12 ± 0.01	0.21 ± 0.01
10	0.05 ± 0.01	0.06 ± 0.01	0.11 ± 0.01
5	0.03 ± 0.01		
2	0.02 ±0.01		

**Table 2:** Main results concerning the fractal structure of silica aerogels: base, neutral and acidic catalysis, composite, sintered and compressed sets.

Synthesis Process	Df	ξ(Å)	a (Å)
Acid set	2.4 ± 0.1	40–320	≈10
Base set	1.8 ± 0.1	40–250	15–20
Neutral set	2.2 ± 0.1	40–310	≈10
Composite set	2.3 and 1.6 ± 0.1	50–5000	≈10 and ≈100
Sintered set	2.3–2.7 ± 0.1	70–130	≈10–20
Compressed set	2.3 ± 0.1	50–130	≈10
